# Antitumor efficacy of BAFF-R targeting CAR T cells manufactured under clinic-ready conditions

**DOI:** 10.1007/s00262-020-02614-8

**Published:** 2020-05-25

**Authors:** Zhenyuan Dong, Wesley A. Cheng, D. Lynne Smith, Brian Huang, Tiantian Zhang, Wen-Chung Chang, Xiuli Wang, Stephen J. Forman, Larry W. Kwak, Hong Qin

**Affiliations:** 1 Toni Stephenson Lymphoma Center, Department of Hematology and Hematopoietic Cell Transplantation, Beckman Research Institute of City of Hope, Duarte, CA 91010 USA; 2 Center for CAR T Cell Therapy, Department of Hematology and Hematopoietic Cell Transplantation, Beckman Research Institute of City of Hope, Duarte, CA 91010 USA

**Keywords:** CAR T cell therapy, Acute lymphoblastic leukemia, GMP production, BAFF-R

## Abstract

B-cell malignancies can potentially be cured by CD19 chimeric antigen receptor (CAR) T-cell therapy. Although clinical response rates can be up to 93% in acute lymphoblastic leukemia, treatment-related antigen loss and lack of therapeutic persistence contribute to disease relapse. These shortcomings of current CAR T-cell therapy indicate the need for biologically relevant target selection and for improving the efficacy and persistence of the CAR T cells, which we have addressed by developing a novel B-cell activating factor receptor (BAFF-R) CAR T-cell therapy with improved therapeutic persistence. BAFF-R is a B-cell survival receptor and highly expressed in B-cell malignancies. We developed a prototype CAR T cell that efficiently and specifically eliminated BAFF-R expressing human B-cell tumors in several xenogeneic mouse models, including models of CD19 antigen loss. We proceeded with translational development and validation of BAFF-R CAR T cells produced under current good manufacturing practices (cGMP). cGMP-grade BAFF-R CAR T cells underwent in vitro and in vivo validation in established models to confirm that the potency and efficacy of our original research modeling was replicated. Food and Drug Administration required release testing was performed to ensure our BAFF-R CAR T cells meet specifications for new drug products. Completing and exceeding these requirements, the data fully support the initiation of a first-in-human Phase 1 trial for BAFF-R-positive relapsed/refractory (r/r) B-ALL.

**Electronic supplementary material:**

The online version of this article (10.1007/s00262-020-02614-8) contains supplementary material, which is available to authorized users.

## Introduction

Chimeric antigen receptor (CAR) T-cell therapy has matured with the recent Food and Drug Administration (FDA) approval of CD19 CAR T cells marking a new era in cancer immunotherapy. This has curative potential for patients with B-cell malignancies, particularly acute lymphoblastic leukemia (ALL) where response (CR) rates of 62–93% were observed [1]. Encouraging results have also been seen in recent clinical trials in non-Hodgkin lymphoma (NHL) patients (CR 23–55%) [2]. However, the potential of CD19 CAR T cells to treat B-cell malignancies is tempered by treatment-related antigen loss and lack of therapeutic persistence that results in disease relapse [3–5]. These shortcomings of current CAR T-cell therapy indicate the need for biologically relevant target selection and for improving the efficacy and persistence of the CAR T cells.

In order to address this need, we have developed a novel B-cell activating factor receptor (BAFF-R) CAR T cell with overall improved therapeutic persistence [6]. BAFF-R is a well-studied B-cell survival receptor that is also highly expressed in B-cell malignancies [7–9]. We have described the development of a specific, high affinity humanized monoclonal antibody against natively expressed BAFF-R, which has limited off-target effects [10]. This was used to develop a prototype CAR T cell capable of efficiently and specifically eliminating BAFF-R expressing human B-cell tumors in several xenogeneic mouse models, which notably included models of CD19 antigen loss [6]. Herein, we report the translational development and validation of BAFF-R CAR T cells produced under current good manufacturing practices (cGMP) intended for use in a first-in-human Phase 1 trial for BAFF-R-positive relapsed/refractory (r/r) B-ALL.

## Methods

*CAR T*-*cells*—BAFF-R CAR T cells were produced from peripheral blood mononuclear cells of healthy donors provided by the Michael Amini Transfusion Medicine Center at City of Hope (IRB: 15283) following two previously established protocols: (1) *Research-grade CAR T cells* [6] were produced from activated naïve T cells (T_N_), transfected at MOI = 1, and FACS enriched for CAR-positive T cells (≥ 95%). (2) *cGMP-grade CAR T cells* [11] were produced from CliniMACS-isolated early stage T cells (T_N/MEM_), activated, and transfected with the clinical vector at MOI = 0.5–2. Each batch of isolated donor T cells were divided into two aliquots: (1) CAR T-cell production; and (2) non-transduced T-cell controls (cultured and expanded in parallel to CAR T cells).

*In vitro assays*—Malignant human B-cell lines (Raji, Nalm-6, and Z-138) were purchased from the American Type Culture Collection or Deutsche Sammlung von Mikroorganismen und Zellkulturen GmbH. Modified cell lines (Nalm-6-BAFF-R-KO and Nalm-6-CD19-KO) were previously developed with CRISPR technology [6]. *Cytotoxic T lymphocyte (CTL) assay:* Chromium-51 (^51^Cr) release was used to calculate specific lysis of tumor cells by CAR T cells as previously described [6]. Briefly, ^51^Cr labeled target cells were coincubated with CAR T cells. Released ^51^Cr was detected in clarified supernatant by gamma counter and calculated as a percentage of maximum release. Statistics: mean ± SD of triplicate samples from a single T-cell donor shown; paired Student’s *t* test of experimental versus controls; experiment repeated with at least three different donor T cells. *Degranulation and cytokine release assay:* FACS analysis of CD107a-positive (degranulated) CAR T cells and INF gamma release by CAR T cells in response to tumor were assessed as previously described [6].

*In vivo modeling*—NOD *scid* gamma (NSG) mice were purchased from The Jackson Laboratory and maintained at the Animal Resource Center of City of Hope in accordance to Institutional Animal Care and Use Committee guidelines (IACUC: 15020). NSG mice were challenged (IV) with previously established, luciferase-expressing tumor models followed by treatment with BAFF-R CAR T cells [6]. Tumor progression was monitored by bioluminescent imagining techniques. Briefly, *n* = 5 mice per group were challenged; minimal lethal dose and CAR infusion day in this study were 5 × 10^4^ Z-138, 7 d; and 0.2 × 10^6^ Nalm-6-CD19-KO, 10 d. A single infusion of 1–2 × 10^6^ BAFF-R CAR T cells were administered (IV). Survival data are reported in Kaplan–Meier plots and analyzed by log-rank tests.

## Results

**Fig. 1:**
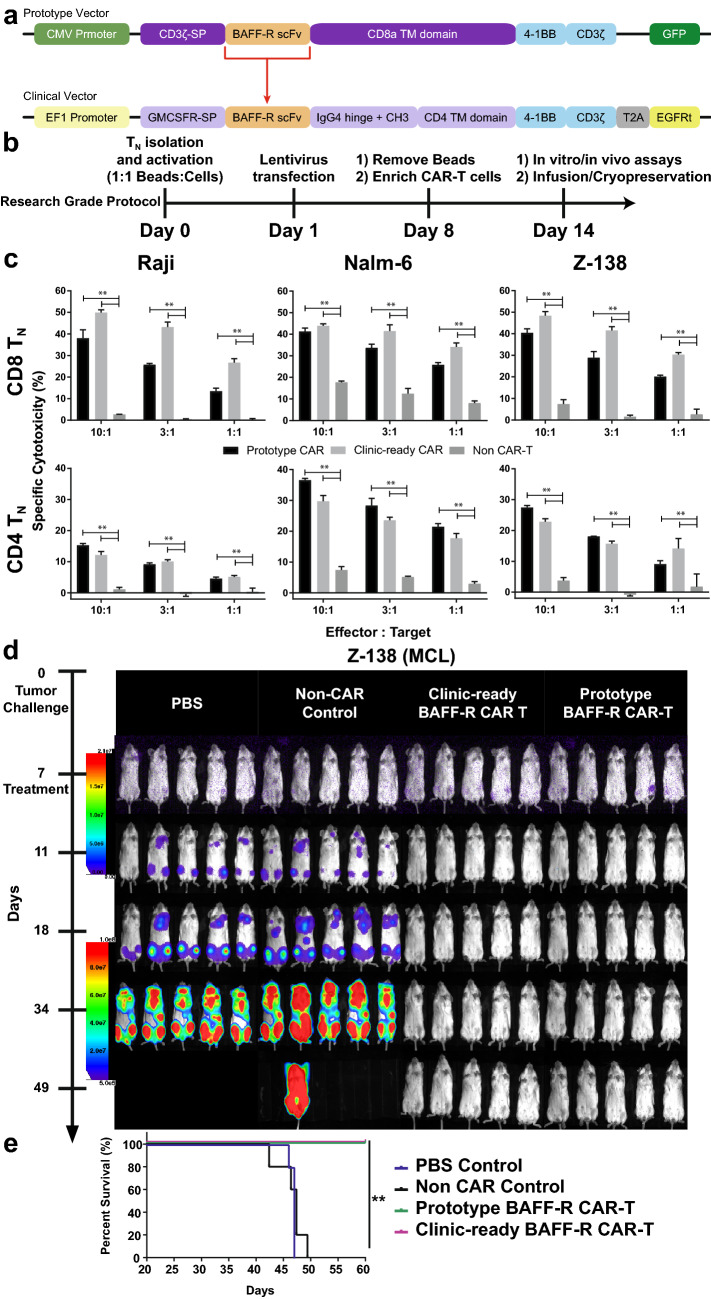
Prototype BAFF-R CAR translated to clinic-ready CAR with equivalent potency. **a** Diagram depicts BAFF-R scFv from research prototype vector (upper) engineered onto clinical vector (lower). Clinical vector was used previously in FDA-approved CD19 CAR T-cell clinical trials. Notable modifications found on the clinical vector include the 4-1BB and CD3ζ motifs, a mutant human IgG4 hinge, with CD3 and truncated EGFRt extracellular motifs expressed via T2A sequence. **b** Timeline outlines the critical steps in research-grade CAR T-cell production from naïve T cell (T_N_) with CAR-positive T-cell enrichment. CAR T cells in Fig. 1 were produced according to this protocol. **c** Graph of calculated specific cytotoxicity following a cytotoxic T lymphocyte assay of CD4 or CD8 T_N_ BAFF-R CARs against various B-cell malignancies. Prototype or clinic-ready CAR T cells (effectors) were coincubated with ^51^Cr labeled B-cell tumors (target) at indicated effector to target ratios. Non-transduced T cells were used as controls. Two-way ANOVA and multiple comparisons test: **P < 0.01 versus non-CAR control. **d** Bioluminescent imaging shows Z-138 (MCL) in vivo luciferase-expressing, tumor model following treatment with BAFF-R CAR T cells. Mice challenged with tumors at day 0 were randomized and treated with a single infusion of either 1 × 10^6^ prototype or clinic-ready BAFF-R CAR T cells at day 7. Non-transduced T cell or PBS was used as controls. **e** Mice were monitored for up to 60 days depicted on the survival curve. Log-rank test: **P < 0.01 Clinic-ready and Prototype BAFF-R-CAR versus controls

We elected to employ a proven clinical development strategy already in use for CAR T-cell production for patients at City of Hope [11–13]. To create the clinical-grade vector BAFF-R:4-1BB:ζ/EGFRt, the BAFF-R-targeting single-chain variable fragment (scFv) [10] was cloned into a second-generation pHIV7 clinical lentiviral vector backbone (Fig. 1a), containing the 4-1BB and CD3ζ motifs, a mutant human IgG4 Fc hinge and CD3 extracellular motif and a truncated EGFR (EGFRt) extracellular motif (see Supplementary Table 1). The latter replaces the GFP tracker from the prototype vector (BAFF-R:4-1BB:ζ/GFP in a pLenti7.3/v5-DEST lentiviral vector backbone), and can be used as a suicide switch to mitigate cytokine release syndrome (CRS) caused by over-activated CAR T cells [14]. Following the research-grade CAR production protocol (Fig. 1b), the prototype and clinic-ready (clinical vector used in research-grade production) BAFF-R CAR T cells were produced as previously described [15] for a head-to-head in vitro and in vivo comparison to verify that CAR T cells produced using the two vectors were equivalent. The research-grade production run yielded ≥ 90% enriched naïve T cell (T_N_)-derived prototype or clinic-ready CAR T cells, measured by FACS analysis of CD3 and tracker (GFP or EGFRt), respectively, and equivalent expansion rates were observed (Supplementary Figure S1a).

We compared specific cytotoxicity of both CD4 and CD8 T_N_-enriched CAR T cells produced using clinical vector BAFF-R:4-1BB:ζ/EGFRt and prototype vector BAFF-R:4-1BB:ζ/GFP using an vitro cytotoxic T lymphocyte (CTL) assay [15] in a panel of chromium-51 labeled target malignant B-cell lines including Nalm-6 B-ALL (Fig. 1c). In all tumor models and at varying effector to target ratios, BAFF-R:4-1BB:ζ/EGFRt and BAFF-R:4-1BB:ζ/GFP CAR T cells showed similar in vitro activity. Similarly, CAR T cells generated using the two BAFF-R vectors were compared in an established, benchmark B-cell lymphoma tumor model, Z-138 (Fig. 1d). Tumor bearing mice, randomized prior to a single CAR T-cell infusion, were monitored by bioluminescent imaging of luciferase-expressing Z-138 tumors as previously described [15]. Rapid tumor clearance was observed in both the prototype and clinic-ready BAFF-R CAR treated mice. Mice in these treated groups also demonstrate comparable and significant tumor free survival compared to controls (Fig. 1e). Together, these results show that the BAFF-R:4-1BB:ζ/EGFRt clinical-grade vector-generated CAR T cells are functionally equivalent to those produced by the BAFF-R:4-1BB:ζ/GFP prototype vector. We could then reasonably proceed to further preclinical development.

**Fig. 2:**
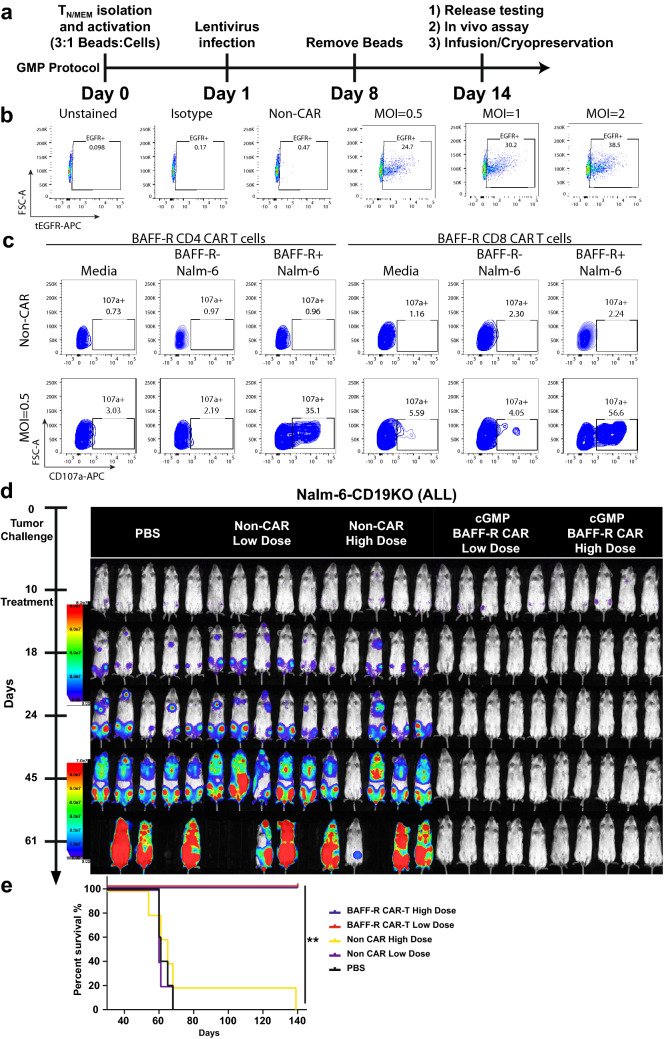
Pilot production of BAFF-R CAR T cells via GMP protocol and release tests. **a** Timeline outlines the critical steps in cGMP CAR T-cell production from early stage T cell (T_N/MEM_) with FDA required release testing. CAR T cells in Fig. 2 were produced according to this protocol. **b** FACS plots show BAFF-R CAR-positive T cell as measured by EGFRt following transfection at various MOIs. CliniMACS isolated T_N/MEM_ cells were transfected at various MOIs and assessed for desired transfection profile, potency, and release requirements. **c** FACS plots of BAFF-R CAR T-cell functional potency as measured by a CD107a degranulation assay. CD4 or CD8 BAFF-R CAR T cells were coincubated with either BAFF-R-positive or negative Nalm-6 B-ALL line. **d** Bioluminescent imaging shows Nalm-6-CD19KO (ALL) in vivo luciferase-expressing tumor model following treatment with BAFF-R CAR T cells. Mice challenged with tumors at day 0 were randomized and treated with a single infusion of low dose 2.8 × 10^6^ T_N/MEM_ or high dose 5.6 × 10^6^ T_N/MEM_, which yielded 1 × 10^6^ and 2 × 10^6^ BAFF-R CAR T cells, respectively, at day 10. Controls were non-transduced T cell or PBS. **e** Mice were monitored for up to 140 days depicted on the survival curve. Log-rank test: **P < 0.01 GMP BAFF-R-CAR High and Low Dose versus controls

We next evaluated cGMP protocols for lentiviral vector production and CAR T cell manufacture with the aim of completing FDA-mandated investigational new drug (IND)-enabling studies following the procedure outlined in Fig. 2a. We therefore set out to optimize BAFF-R CAR T-cell production under several key criteria including: minimum viral load; maximized transduction efficiency, functional potency; and passing FDA required release testing.

The City of Hope Biologics & Cellular GMP Manufacturing Center produced BAFF-R:4-1BB:ζ/EGFRt clinical vector under cGMP conditions. Transfection of T_N/MEM_ cells obtained from healthy donors [Michael Amini Transfusion Medicine Center at the City of Hope (IRB: 15283)] was evaluated at three multiplicities of infection (MOI), 0.5, 1, and 2 (Fig. 2b). All three MOIs produced viable CAR T cells with equivalent growth and expansion rates (Supplementary Figure S1b), and transduction efficiency measured by FACS analysis of EGFRt were equivalent for all MOIs. Maximized functional potency was confirmed by BAFF-R specific activation of BAFF-R CAR T cells, measured by CD107a degranulation marker following incubation with target cell lines (Fig. 2c, Supplementary Figure S2) as previously described [15]. Functional potency at MOI = 0.5 was similar to MOI = 1 or 2. Furthermore, the BAFF-R-induced CAR T cell INF-γ release did not vary significantly between MOIs (Supplemental Figure S3). Subsequent batches of BAFF-R CAR T cells were produced with MOI = 0.5 transfection and underwent FDA required release testing.

Three donor leukapheresis products were successfully used to generate BAFF-R CAR T cells following the GMP protocol derived from a successful production strategy that implements CliniMACS automation for T-cell isolation [11]. The original manufacturing strategy was developed and implemented at City of Hope and produces CAR T cells from early stage T cells (T_N/MEM_) consisting of naïve, memory-like stem, and central memory T cells. Each batch of cells were closely monitored during the transfection, expansion, and activation stages. Each exceeded the minimum requirements of qualification release testing set forth by the FDA, which included viability (≥ 70%), identity measured by CD3 (≥ 80%), transduction efficiency and CAR expression measured by EGFRt (≥ 10%), WPRE copy insertion (≤ 5 copies/cell), and VSVG copy insertion (≤ 2.5 copies/50 ng genomic DNA) (Table 1).

**Table 1:** Qualification release testing

Culture condition	MOI	Viability (%)	Identity (CD3) (%)	Transfection efficiency (tEGFR) (%)	WPRE (Copy Number/Cell)	VSVG (Copy Number/50 ng gDNA)
HD409 (Run 1)						
Plate	Mock	87	99.9	0.47	0.0023648	< 2.5 copies
Plate	0.5	86	99.9	24.7	0.8429023	< 2.5 copies
Plate	1	84	99.9	30.2	0.9550843	< 2.5 copies
Plate	2	86	99.9	38.5	1.2226313	< 2.5 copies
HD408 (Run 2)						
Bag	Mock	83	99.7	0.57	0.0046748	< 2.5 copies
Bag	0.5	79	99.8	30.9	1.2712262	< 2.5 copies
Plate	Mock	85	99.7	0.68	0.0061125	< 2.5 copies
Plate	0.5	81	99.7	35.5	0.9591207	< 2.5 copies
HD562 (Run 3)						
Bag	Mock	94	99.8	0.85	0.002598089	< 2.5 copies
Bag	0.5	91	99.8	24.5	0.556451478	< 2.5 copies

Lastly, we repeated an in vivo study to confirm the efficacy of the BAFF-R:4-1BB:ζ/EGFRt CAR T cells produced under the GMP protocol, as the research and GMP production strategies differed in the specific T-cell isolation and expansion steps. Specifically, the GMP strategy isolates all early stage T cells (naïve, memory-like stem, and central memory), whereas the research product was exclusively produced from naïve T cells. Secondly, the CAR T-cell enrichment was omitted in the GMP procedures, yielding a more heterogeneous product consisting of 25–35% CAR T cells. Therefore, experimental group dosing in the in vivo Nalm-6 ALL tumor model study is denoted as low dose (2.8 × 10^6^ T cell yielding an effective 1 × 10^6^ BAFF-R CAR T cells) or high dose (5.6 × 10^6^ T cell yielding an effective 2 × 10^6^ BAFF-R CAR T cells) (Fig. 2d). Remarkably, mice treated with either a low or high dose of cGMP BAFF-R CAR T cells demonstrated rapid tumor clearance and tumor-free survival up to 140 days after tumor challenge (Fig. 2e).

## Discussion

Rapid translation of BAFF-R CAR T-cell therapy to clinical application is warranted because it is a potentially promising immunotherapy, especially for antigen-loss r/r B-ALL following CD19-targeted treatments. To expedite this process we used a proven platform for CAR gene delivery and CAR T-cell production in order to ensure reliability and rapid translation into an upcoming first-in-human clinical trial. Notably, the original prototype BAFF-R CAR T cells were produced with a highly selective cell isolation and enrichment protocol that yielded a nearly homogeneous (> 90%) population BAFF-R CAR naïve T cells. This research protocol modeled the ideal early stage T cell for CAR T-cell production and ensured reproducibility of results particularly for in vivo dosing. However, it would be challenging to reliably produce and deliver enriched naïve CAR T cells from patients in the clinic. The chosen platform seeks to balance proven efficacy seen in the preclinical studies with FDA-approved, GMP protocols. BAFF-R CAR T cells intended for patients will therefore include all early stage T cells following the T_N/MEM_ GMP protocol [13]. Although we are not enriching BAFF-R CAR-positive cells prior to infusion, the cell product has consistently been 24–35% CAR-positive; exceeding the FDA requirements of > 10%.

Collectively, the data fully support the initiation of a first-in-human Phase 1 clinical trial to examine the safety of BAFF-R CAR T cells, particularly given the urgent need to address r/r B-ALL following CD19-targeted immunotherapy. We expect a comparable safety profile of our BAFF-R CAR T cells to other CARs in clinical use, and would also expect to observe efficacy based on the strong preclinical results.

### Electronic supplementary material

Below is the link to the electronic supplementary material.

Supplementary material 1 (PDF 468 kb)

Supplementary material 2 (PDF 534 kb)

## Data Availability

Data and materials produced in this study are protected by City of Hope intellectual property patents but will remain available to qualified investigators at other research organization by establishing a Material Transfer Agreement (MTA). MTA requests should be directed to the corresponding author.
