# Supplementary material for: Antitumor efficacy of BAFF-R targeting CAR T cells manufactured under clinic-ready conditions
Source: Cancer Immunol Immunother. 2020 May 25;69(10):2139–45. doi: 10.1007/s00262-020-02614-8 (PMC7511472; doi:10.1007/s00262-020-02614-8)
Supplement: Supplementary file 1 — Supplementary material 1 (PDF 468 kb) [file 262_2020_2614_MOESM1_ESM.pdf]

Supplementary Figure Legends

Supplementary Figure S1. BAFF-R CAR T cell expansion. Growth curve plots the expansion of BAFF-R CAR T cells produced by our **a** Research Grade and **b** cGMP protocols. **a** T_N cells were transfected with clinical or prototype vectors and stimulated with varying stimulation beads:cell ratios. Expansion was observed one week post CAR-T cell enrichment. **b** $T_{N/MEM}$ cells were transfected with clinical vectors at MOI= 0.5, 1, or 2. CAR T cells were cultured and monitored for 14 days. Non-transduced T cells were used as a control.

Supplementary Figure S2. cGMP BAFF-R CAR T cell degranulation. FACS plots of BAFF-R CAR T cell from Supplemental Figure 1 show **a** gating strategy and **b** functional potency as measured by a CD107a following a degranulation assay. CD4 or CD8 BAFF-R CAR T cells were coincubated with either BAFF-R-positive or –negative Nalm-6 B-ALL line.

Supplementary Figure S3. cGMP BAFF-R CAR T cell IFN-γ release assay. Graph shows ELISA measurements of IFN-γ released by BAFF-R CAR T cell from Supplemental Figure 1. cGMP BAFF-R CAR T cells were coincubated with either BAFF-R-positive or –negative Nalm-6 B-ALL line. IFN-γ was measured from the cell supernatant.

Supplementary Table 1. Vector and CAR T cell Nomenclature

	Nomenclature	*Description*
Lentiviral vector	prototype vector	BAFF-R:4-1BB:ζ/GFP in a pLenti7.3/v5-DEST lentiviral vector backbone
	clinical vector	BAFF-R:4-1BB:ζ/EGFRt in a second-generation pHIV7 clinical lentiviral vector backbone
CAR-T	Prototype CAR-T	Prototype vector used in research-grade production
	Clinic-ready CAR-T	Clinical vector used in research-grade production
	cGMP CAR-T	Clinical vector used in GMP production
