# Supplementary material for: Antitumor efficacy of BAFF-R targeting CAR T cells manufactured under clinic-ready conditions
Source: Cancer Immunol Immunother. 2020 May 25;69(10):2139–45. doi: 10.1007/s00262-020-02614-8 (PMC7511472; doi:10.1007/s00262-020-02614-8)

Supplementary Figure S1

a

Research Grade BAFF-R CAR T-cell Expansion

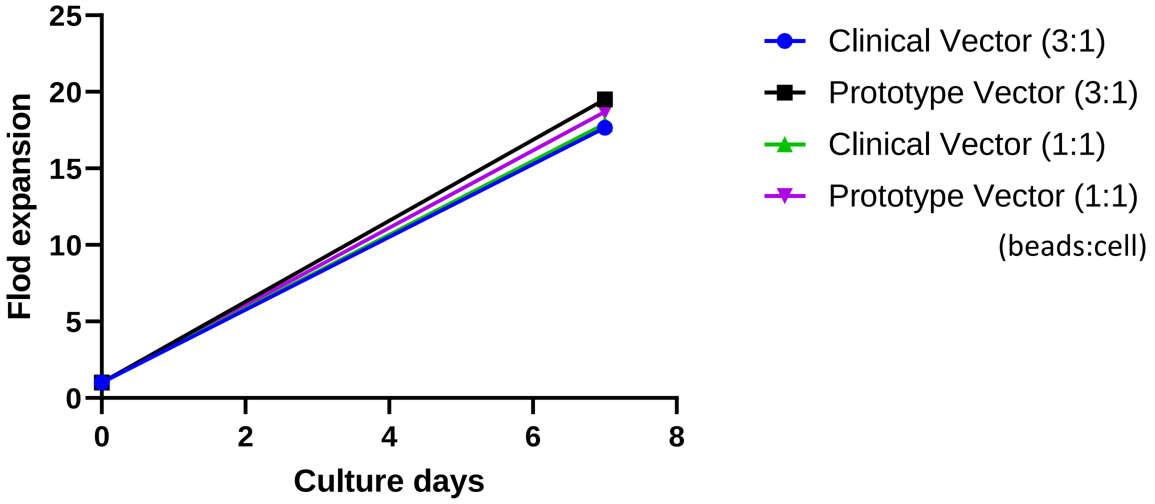

b

cGMP Grade BAFF-R CAR T-cell Expansion

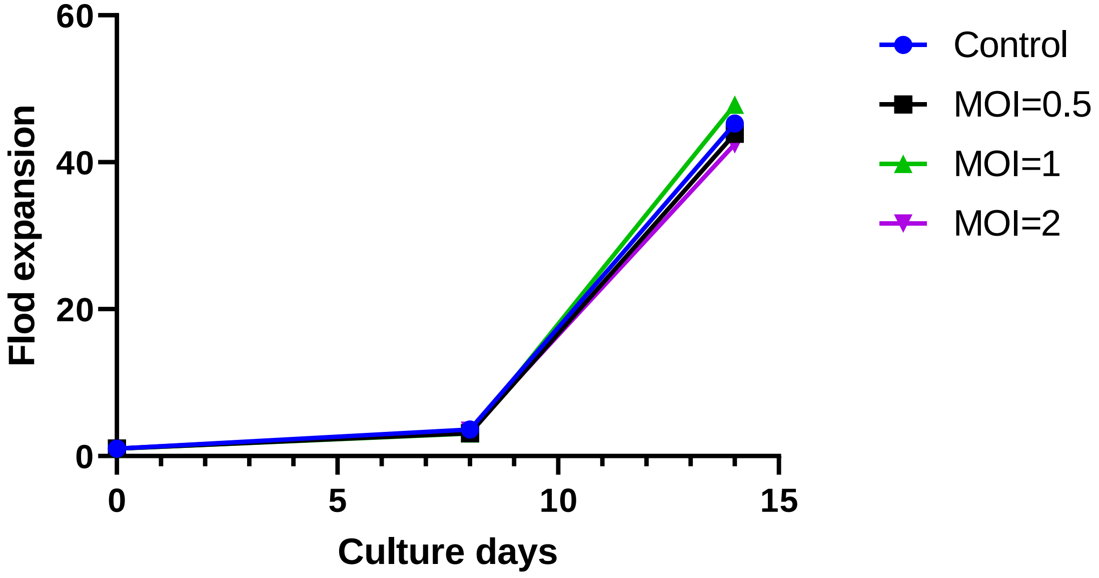

Supplementary Figure S2

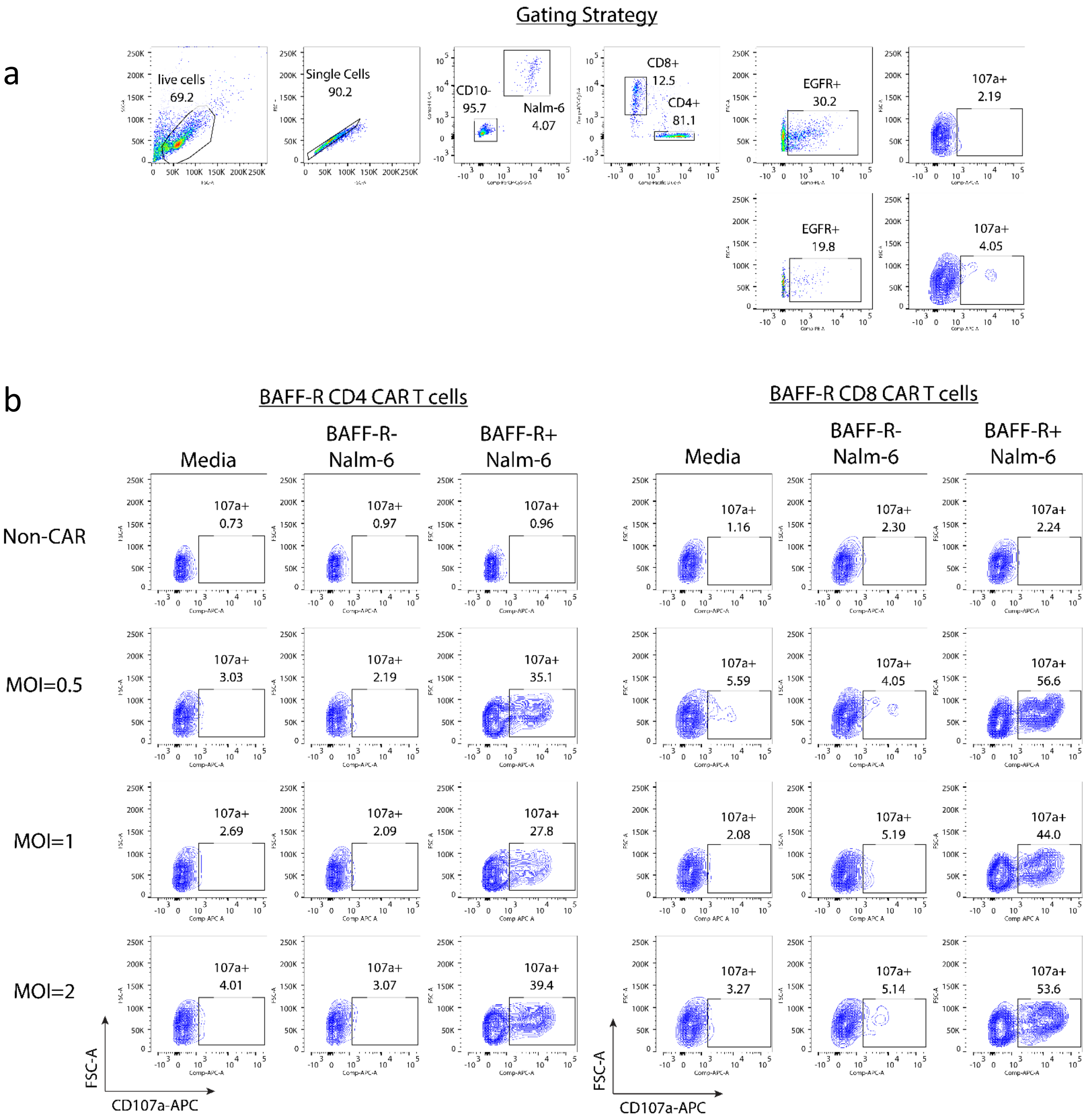

Supplementary Figure S3

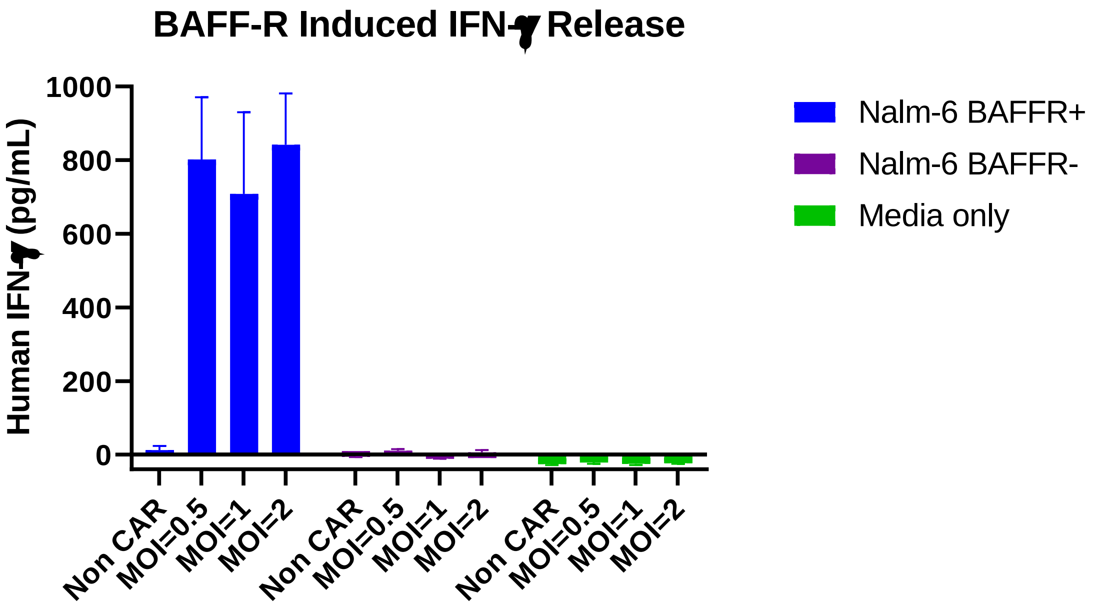

Supplement: Supplementary file 2 — Supplementary material 2 (PDF 534 kb) [file 262_2020_2614_MOESM2_ESM.pdf]
